# Population Status of a Regionally Endangered Plant, *Lunaria rediviva* (Brassicaceae), near the Eastern Border of Its Range

**DOI:** 10.3390/biology12060761

**Published:** 2023-05-23

**Authors:** Anatoliy A. Khapugin, Gennadiy G. Chugunov

**Affiliations:** 1 Joint Directorate of the Mordovia State Nature Reserve and National Park “Smolny”, 430005 Saransk, Russia; 2 Institute of Environmental and Agricultural Biology (X-BIO), Tyumen State University, 625003 Tyumen, Russia; 3 Department of General Biology and Ecology, Mordovia State University, 430005 Saransk, Russia

**Keywords:** ontogenetic structure, density, morphometrics, reproductive biology, threatened species, weather conditions

## Abstract

**Simple Summary:**

This paper is devoted to the study of the *Lunaria rediviva* population at the eastern border of its range (National Park “Smolny”, Republic of Mordovia, Russia) in 2013–2018. Ontogenetic structure of the population has been identified by distinguishing juvenile, mature vegetative, and reproductive individuals. We found changes in the ontogenetic structure of the population from 2013 to 2018. The type of its population changed from vegetatively oriented to bimodal, with a decrease in proportion of mature vegetative individuals. We found a significant negative correlation between the fruit set and the moisture in mid-July, and wind strength in late May and early June. It was found that the number of both flowers and fruits per individual is significantly positively correlated with the precipitation in late April, and they are negatively correlated with these parameters and the temperature in late July. We assume that the habitat shading negatively influences the *L. rediviva* population status.

**Abstract:**

Long-term studies of plant populations provide valuable knowledge on the influence of various environmental factors on plant species. The status of edge-range species populations is especially important to be studied due to their higher vulnerability to extinction. This paper aimed to study the *Lunaria rediviva* population at the eastern border of its range (National Park “Smolny”, Republic of Mordovia, Russia). The study was carried out in 2013–2018. Assessment of the *L. rediviva* population was performed on the basis of individual parameters of plants (height of the individual, number of leaves per individual, number of inflorescences, flowers, fruits per one generative individual, and the fruit set), and density of individuals. Ontogenetic structure of the population was identified by distinguishing juvenile, mature vegetative, and reproductive individuals. The relationships between weather conditions (mean values of temperature, air moisture, wind strength, precipitation divided to three decades per month) and population parameters of *L. rediviva* were identified. Results showed changes in the ontogenetic structure of the population. The type of its population changed from vegetatively oriented to bimodal, with a decrease (R^2^ = 0.686) in the proportion of mature vegetative individuals. We demonstrated a significant decline in some parameters of the *L. rediviva* reproduction. We found a significant negative correlation between the fruit set and the moisture at mid-July (r = −0.84, *p* < 0.05), and wind strength in late May (r = −0.83, *p* < 0.05) and early June (r = −0.83, *p* < 0.05). It was found that the number of both flowers and fruits per individual is significantly positively correlated with the precipitation in late April, and they negatively correlated with these parameters and the temperature in late July. We assume that the habitat shading negatively influences the *L. rediviva* population status.

## 1. Introduction

Although we can see great progress in understanding the causes of the global biodiversity loss, major knowledge gaps still remain. Among these gaps are an uncertainty of how many species are threatened at global, national, and regions levels [1], a lack of data on the impacts of the biodiversity loss on habitat features and species communities, and geographic and taxonomic biases in the available information [2]. To develop a conservation strategy for threatened plant species, the first step includes assessment of conservation status by applying categories and criteria of the IUCN Red List of Threatened Species [3,4,5,6]. It is especially important for species, which are not listed in the Global IUCN Red List. National and regional Red Lists play a valuable role in informing global conservation efforts [7], although there is still a gap between national and global assessor [8,9]. In situ conservation measures are the best methods of biodiversity protection [10]. Of them, Protected Areas are recognised as the best tool for biodiversity and habitat conservation on global scale [11,12].

In various regions of the world, one of the most relevant drivers of species extinction is habitat fragmentation, a worldwide phenomenon recently considered one of the major threats to plant populations [13,14,15] and entire species. Habitat fragmentation often causes a decrease in the size and density of populations, which is reflected in their inclusion in Red Data Books [5,16] or assessing under threatened IUCN Red List categories [17]. Some authors [12,18] also showed that various Protected Area types successfully contribute to the preservation and restoration of plant and animal populations in various regions of the world [19,20]. They serve as survival “islands” for populations of edge-range plant species, which are often smaller and less dense than populations in the centre of the range [21,22], and tend to be less viable and more prone to extinction [21,23]. Based on the abundant-centre model, the population abundance of a species is highest at the centre of its range, and it is decreasing towards the range edges [24,25]. However, some other studies showed that edge-range populations are not less [26] or even more [27] productive compared to ones in the centre of the range.

In the Republic of Mordovia, a region of European Russia, 164 vascular plants are considered threatened, being included in the regional Red Data Book [28]. Then, they were estimated according to the IUCN Red List categories and criteria [29]. It demonstrated the presence of 68 Critically Endangered, 37 Endangered, 21 Vulnerable, 31 Near-Threatened, and 6 Data-Deficient-evaluated vascular plant taxa in the flora of the Republic of Mordovia. This paper is aimed to assess the population status of *L. rediviva*, a Tertiary relict perennial regionally Endangered species, at the eastern border of its range (Republic of Mordovia, European Russia). We addressed the following questions: (i) What is the status of *L. rediviva* population at the eastern border of its range? (ii) How do weather conditions influence the state of *L. rediviva* in the study area?

## 2. Materials and Methods

### 2.1. Study Species

*Lunaria rediviva* L. (Brassicaceae), a Tertiary relict perennial species distributed from Italy and former Yugoslavia (in the south) to the Baltic Sea and Sweden (in the north), and from Portugal (in the west) to the Volga River (in the east) [30,31,32]. It is one of three representatives of this European genus, though having the largest distribution relative to the two other ones. *Lunaria rediviva* has been introduced to North America, Great Britain, and Sardinia island [32]. Despite the large natural range in Europe, there is a lack of publications devoted to the population status of *L. rediviva* and factors threatening it, while most of them are in Russian [33,34,35]. This European species inhabits broadleaved forests, and is considered a threatened plant in Eastern Europe, namely some regions of European Russia [15], Belarus [36], and Ukraine [37]. *Lunaria rediviva* is included in the Red Data Book of the Republic of Mordovia [28] and has recently been assessed as a regionally Endangered species in the Republic of Mordovia, European Russia [29].

*Lunaria rediviva* is a long-lived perennial plant up to 100–140 cm, which has erect stems. Bottom leaves are opposite, but the upper leaves of mature individuals are alternate. The bottom leaves are ovate with pointed tips and dentate margins. The intermediate leaves are cordate with spinulose-dentate margin, on long petioles. The upper leaves are ovate-acuminate, on short petioles. The inflorescence is panicle and it includes apical and lateral racemes. Each raceme often has from 3 to 18 (sometimes more) flowers. Petals are to 20 mm long, lilac to violet. Siliculae are 35–90 × 15–35 mm, elliptical, rarer ovate-elliptical or elongate-elliptical. Each fruit produces 2–8 seeds [30,33,34].

In the Republic of Mordovia, the population of *L. rediviva* is located near the eastern edge of its range (Appendix A). It is preserved in forest communities of Protected Areas, including National Park “Smolny”. *Lunaria rediviva* is included in the Red Data Book of the Republic of Mordovia [28]. In National Park “Smolny”, *L. rediviva* was found for the first time in 2006 [38]. A preliminary study of this population was carried out only in 2008. It was revealed that this population only contains juvenile and generative individuals [39], which looks to be erroneous.

### 2.2. Field Studies

**Figure 1:**
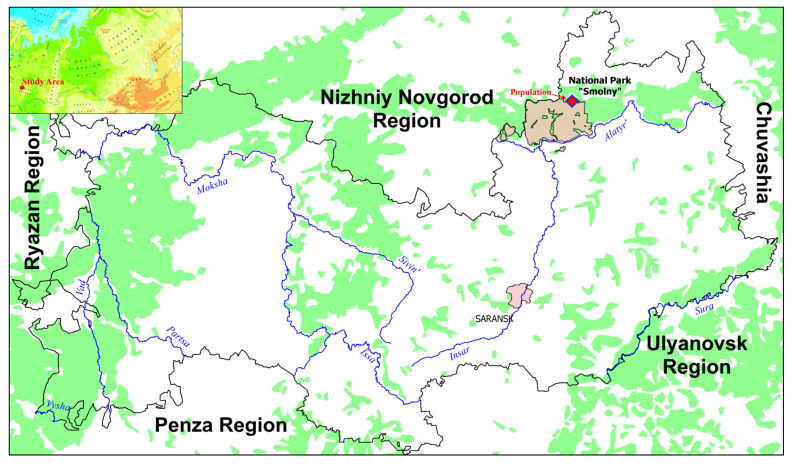
Geographical location of a *Lunaria rediviva* population studied in the National Park “Smolny” (European Russia). Map with modifications from https://www.eea.europa.eu/data-and-maps/figures/physical-map-of-eurasia (accessed on 18 May 2023).

**Figure A1:**
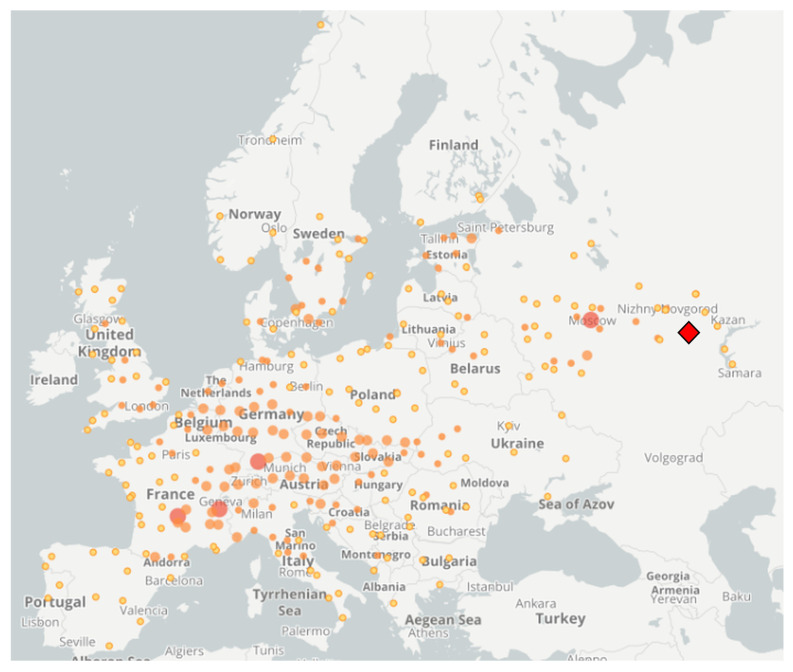
The position of the study area (National Park “Smolny”; marked by red diamond) within the natural range of *Lunaria redivia* in Europe. Source: the map was exported from GBIF depository (https://www.gbif.org/, accessed on 20 April 2023).

**Figure A2:**
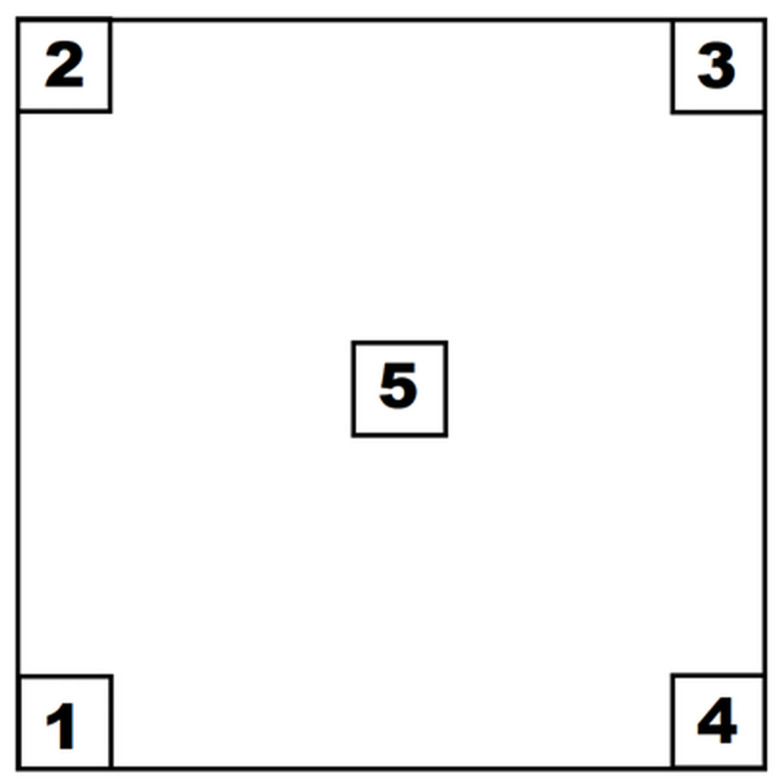
The scheme of 1 m × 1 m plots (designated by numbers 1–5) positions within a large (5 m × 5 m) plot for investigating the *Lunaria rediviva* population near the eastern edge of its range (European Russia).

In August 2013–2018, field investigations were carried out in National Park “Smolny” (54.874840° N, 45.513740° E; Figure 1 and Figure A1). For this purpose, we established one large (5 m × 5 m) plot, with five square plots (1 m × 1 m), as it is shown in Figure A2. In each plot, all individuals were counted and measured. Assessment of the *L. rediviva* population status was performed based on the following morphological parameters of individuals: height of the individual, number of leaves per individual, numbers of inflorescences, flowers, fruits per one generative individual, and the fruit set. The density (number of individuals per 1 m^2^) of *L. rediviva* per 1 m × 1 m plot was calculated. The population type was identified based on [40].

Based on morphometric data, we distinguished three ontogenetic classes of *L. rediviva* individuals, namely juvenile (j), mature vegetative (v) (non-flowering adults), and reproductive (g) (flowering adults) according to [33,34]. Comparison of individuals’ parameters for the *L. rediviva* populations has been carried out for each ontogenetic class.

To reveal weather conditions influencing the *L. rediviva* conditions, climatic data were extracted from https://rp5.ru/ (accessed on 15 April 2023) for the nearest meteorological station (Alatyr, 54.819900° E, 46.581300° E) for 2013–2018. We used four weather parameters (temperature, air moisture, wind strength, precipitation). We calculated mean values for each decade in a month based on daily values of weather parameters.

### 2.3. Data Analysis

Statistical analysis was performed using PAST 3.14 [41]. Minitab ver. 18.1 (State College, PA, USA) and Microsoft Excel. The Pearson’s correlation analysis was carried out to find relationships between weather conditions of the ongoing year and population parameters of *L. rediviva* population. Linear regression was used to determine which weather factors were potentially influencing parameters of the *L. rediviva* population. In all regressions, weather parameters were independent variables and the population parameters were dependent variables.

## 3. Results

**Figure 2:**
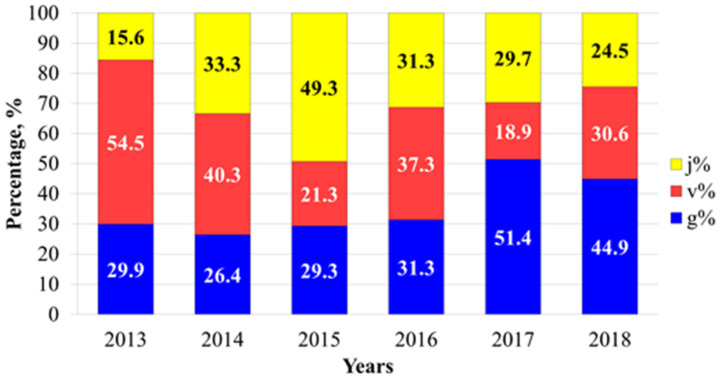
Dynamics of the ontogenetic structure of the *Lunaria rediviva* population in the Republic of Mordovia (Russia) in 2013–2018. Designation of ontogenetic classes: j%—juvenile individuals, v%—mature vegetative individuals, g%—reproductive individuals.

Near the eastern edge of the natural range, the population of *L. rediviva* is vegetatively oriented sensu [40]. In the ontogenetic spectrum, non-flowering individuals (juvenile and mature vegetative plants) dominate (Figure 2). Just in 2017 and 2018, the *L. rediviva* population was characterised as bimodal, when the numbers of reproductive and vegetative individuals were approximately the same.

**Figure 3:**
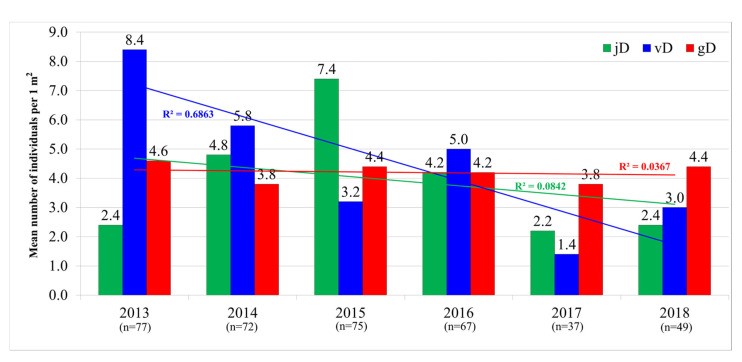
The density of *Lunaria rediviva* individuals per each ontogenetic class in 2013–2018 at the eastern edge of its range (Republic of Mordovia, Russia). Designations: jD—density of juvenile individuals, vD—density of mature vegetative individuals, gD—density of reproductive individuals; n—the total number of individuals counted in the study area per year.

Accordingly, with an increase in the proportion of reproductive individuals in the ontogenetic structure in 2017, the density of non-flowering plants was the lowest in this year (Figure 3). The mean density of reproductive individuals was stable (R^2^ = 0.0367) during the study period, while we found the decrease in mature vegetative individuals (R^2^ = 0.6863) with the lowest value in 2017 and a slight increase in 2018. During 2013–2018, the density of juvenile individuals increased considerably up to the maximum (7.4 individuals per 1 m^2^) in 2015, with consequent decrease to the same value (2.4 individuals per 1 m^2^) in 2018.

**Figure 4:**
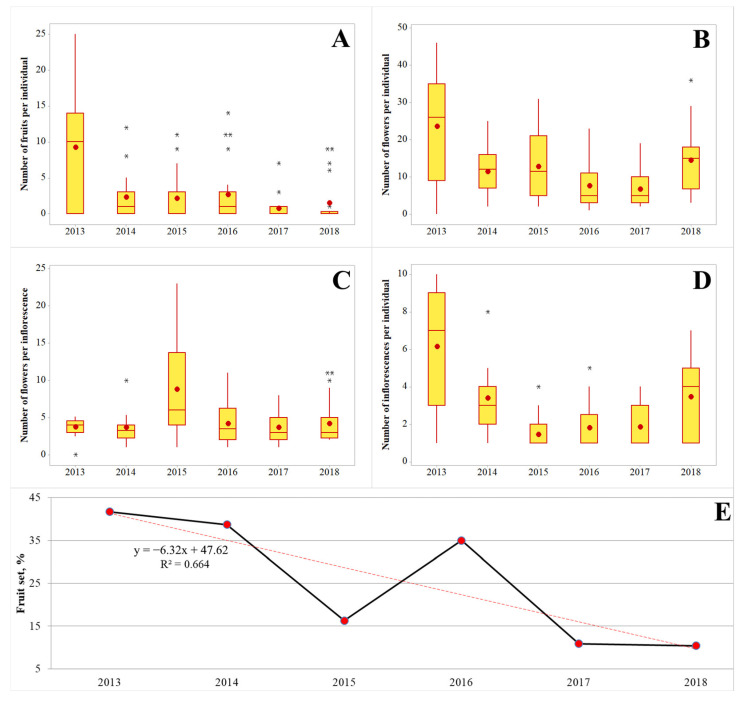
Characteristics of the reproduction for the *Lunaria rediviva* population at the eastern edge of its range (Republic of Mordovia, Russia). Designations: (**A**)—Number of fruits per individual, (**B**)—Number of flowers per individual, (**C**)—Number of flowers per inflorescence, (**D**)—Number of inflorescences per individual, (**E**)—Fruit set calculated as (number of flowers/number of fruits) × 100%. Designations of box-plots: the bottom and top of the boxes are the first and third quartiles; the bands inside the boxes are the median; the red dots inside the boxes are the mean value; whiskers extend to within 1.5 times the interquartile ranges; asterisks beyond the whiskers are outliers. The number of reproductive individuals per year as follows: 2013—23 individuals, 2014—19 individuals, 2015—22 individuals, 2016—21 individuals, 2017—19 individuals, 2018—22 individuals.

The morphological parameters of reproductive individuals changed annually in the studied population (Figure 4). Despite the variation found in the values, we can see that the number of inflorescences, flowers, and fruits per individual in 2018 was considerably lower than it was in 2013. The reasons of this finding are yet to be clarified in further studies, but we can see that the fruit set of the *L. rediviva* population also decreased over six years of the study (Figure 4).

**Figure 5:**
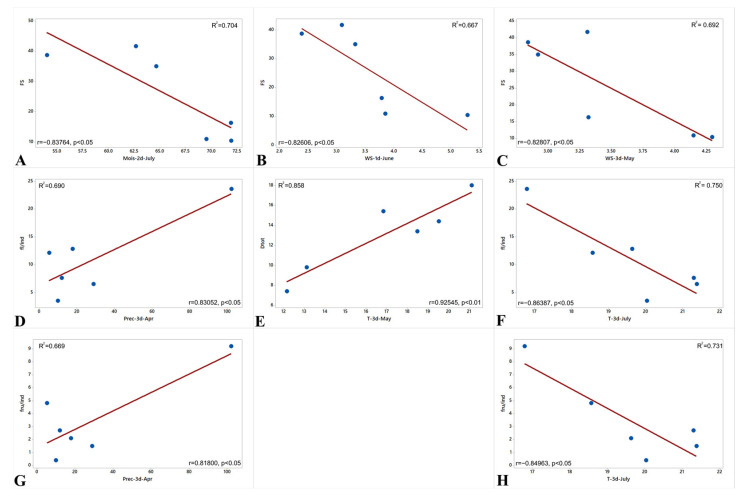
Selected significant relationships between weather conditions and parameters of the *Lunaria rediviva* population at the eastern edge of its range (Republic of Mordovia, Russia). Designations: the fruit set (FS) correlations with (**A**) the precipitation at the second decade of July (Mois-2d-July), (**B**) wind strength at the first decade of June (WS-1d-June), and (**C**) in the third decade of May (WS-3d-May); correlations of the number of flowers per individual (fl/ind) with (**D**) the precipitation at the third decade of April (Prec-3d-Apr) and (**F**) the temperature at the third decade of July (T-3d-July); correlations of the number of fruits per individual (fru/ind) with (**G**) the precipitation at the third decade of April (Prec-3d-Apr) and (**H**) the temperature at the third decade of July (T-3d-July); the correlation of the total density of individuals (Dtot) and the temperature at the third decade of May (T-3d-May) (**E**).

In an attempt to find out the relationships between the decrease in *L. rediviva* reproduction and weather conditions, we conducted a correlation analysis (Figure 5). There was a significant negative relationship between the fruit set and the moisture at mid-July (r = −0.84, *p* < 0.05), and wind strength in late May (r = −0.83, *p* < 0.05) and early June (r = −0.83, *p* < 0.05) (Figure 5A–C). There was a significant positive relationship between the number of both flowers and fruits per individual and the precipitation in late April (Figure 5D,G), while there was a significant negative relationship between these parameters and the temperature in late July (Figure 5F,H). Finally, we found a significant positive relationship (r = 0.93, *p* < 0.01) between the temperature in late May and the total density of *L. rediviva* individuals (Figure 5E).

## 4. Discussion

*Lunaria rediviva* is a threatened, but poorly studied, plant species. There is a lack of data on its population status and environmental preferences. The previous studies in the Tver Region [34] and the Republic of Mordovia [42] were short-term by presenting data about just a few sites within the *L. rediviva* range. Here, we present a long-term study of its population status, preserved within a Protected Area at the east of its range.

In plant populations, the ontogenetic structure is often stable for one species (e.g., *Pyrola chlorantha* [43], *Dracocephalum fruticulosum* [44]) over a certain study period, with some fluctuations. Under unfavourable conditions, the ontogenetic structure of some perennial plants can be changed (e.g., orchids [40], *Pulsatilla vernalis*, see [45]). Consistent with the latter case, we demonstrated the changes in the population type from vegetatively oriented to bimodal (*sensu* [40]) starting since 2017. The changes are also supported by decrease in density of mature vegetative individuals over 2013–2018. We tend to explain these results by shading processes in the studied habitat through the understorey overgrowth, which is consistent with data from the Mordovia State Nature Reserve [35], where it was assumed that the habitat shading by *Acer*-*Ulmus* undergrowth is the most unfavourable factor for *L. rediviva* due to the penetration of grasses from meadow communities adjacent to the *L. rediviva* site. For the Central Forest State Nature Reserve, the decrease in density of *L. rediviva* individuals by 3.1–3.7 times, depending on the site, was demonstrated [34]. To be completely sure, the further special studies of this influence are needed. However, these changes in ontogenetic spectrum can be a part of long-term fluctuations in the ontogenetic structure of *L. rediviva* population.

The pollinators of *L. rediviva* are predominantly bees, bumblebees, and butterflies [33]. This can explain the obtained decrease in the fruit set (Figure 3) with increase in the wind strength, since the wind hinders the insects in pollination. At the same time, drier conditions lead to the fruit set decline at the time of the fruit setting and ripening (second decade of July). The sufficient precipitation amount at early stages of the growing season is important for further plant development [46,47]. This was supported by our findings of the positive contribution of the precipitation in late April (the time of *L. rediviva* seed germination) to the high number of flowers and then fruits in the studied population.

At present, it is difficult to explain reasons of decreasing in the number of fruits with increase in the late-July temperature. We assume that the decline in fruit number may be contributed by the more active development of *Adela rufimitrella* Scopoli, 1763 larvae under higher temperature at the moment of the *L. rediviva* fruit setting. Larvae of this moth are fed by the seeds of Brassicaceae species [33,48]. Further studies are needed to test our assumption.

## 5. Conclusions

The obtained results demonstrate the significant decline in parameters (i.e., fruit set, number of fruits) of the *L. rediviva* reproduction in a population at the eastern edge of its range. This highlights an alarming situation with the population status of this plant species. Taking into account that in various parts of its range *L. rediviva* is characterised by vegetative reproduction, the decline in proportion of mature vegetative individuals may further lead to the change of its regional IUCN Red List status from “Endangered” (at present) to “Critically Endangered”. However, we must note this change refers to the conservation status of *L. rediviva* on the local scale, since at the global scale the taxon is not catalogued. We found that weather conditions impact mainly parameters of reproductive individuals. Although we found the influence of weather conditions on population parameters of *L. rediviva*, we also assume the negative impact of the habitat shading by understorey species overgrowth. Further investigations are proposed in this direction.

## Data Availability

The data presented in this study are available on request from the corresponding author.
